# Ignatzschineria indica Bacteremia Initially Misdiagnosed in a Patient With a Maggot-Infested Wound

**DOI:** 10.7759/cureus.61880

**Published:** 2024-06-07

**Authors:** Andrew Strike, Hossny Alaws, Benjamin Welch, Aleksandra Ignatowicz

**Affiliations:** 1 Internal Medicine, Northeast Georgia Medical Center Gainsville, Gainesville, USA

**Keywords:** emerging pathogens, infectious diseases, pcr testing, cellulitis, gram-negative bacteria, sepsis, maggot infestation, chronic wounds, bacteremia, ignatzschineria indica

## Abstract

Gram-negative bacteremia in hospitalized patients often leads to prolonged hospital stays, increased healthcare costs, and mortality rates. Simultaneously, the presence of comorbidities like chronic wounds increases the risk of severe infection and complicated hospital courses involving amputation, broad-spectrum antibiotic use, and repeat hospital admissions, after discharge. This case presents a 72-year-old male with a past medical history significant for chronic lower extremity cellulitis with multiple prior hospitalizations. On admission, the patient had a chief complaint of progressively worsening left lower extremity pain along with nausea, vomiting, and diarrhea. CT imaging of the left lower extremity suggested severe cellulitis without signs of osteomyelitis. Blood cultures initially suggested *Corynebacterium jeikeium*, but were sent to an outside facility due to ambiguity of results. The outside facility identified the pathogen as *Ignatzschineria indica*. After confirming the results, antibiotics were appropriately de-escalated to oral levofloxacin. The patient continued to show clinical improvement and was discharged with follow-up appointments scheduled for infectious disease and bi-weekly visits to wound care. Considering the increasing prevalence of chronic wounds in the United States, awareness and recognition of emerging pathogens are crucial for the timely diagnosis, treatment, and management of these complex patients. Our case adds to the growing body of reports on the management of *I. indica* bacteremia resulting from maggot-infested wounds.

## Introduction

Gram-negative bacteremia is a common cause of sepsis in the intensive care setting and is associated with poor outcomes, especially in patients with comorbidities [1,2]. These infections are linked to extended hospitalizations, elevated healthcare costs, and mortality rates exceeding 30% [1]. One common comorbidity associated with bacteremia is the presence of chronic wounds [2,3]. *Ignatzschineria* species are an emerging pathogen in North America. *Ignatzschineria indica* is a nonmotile, beta-hemolytic, catalase-positive, oxidase-positive, aerobic, gram-negative bacillus that is part of the *Gammaproteobacteria* class [4]. This species of bacteria is associated with cases involving chronic wounds with maggot infestation [4-7]. Fly larvae serve as a vector through the colonization of open wounds, allowing for proliferation and infection within the host tissue. While still rare, there have been multiple recent cases of cellulitis from *Ignatzschineria* species in the setting of maggot-infested wounds [8]. Patients present with typical cellulitis symptoms: erythema, warmth to touch, elevated inflammatory markers, and purulent discharge [9]. Due to the rarity of this pathogen, it is often misdiagnosed on routine blood cultures. Laboratory methods often require 16S rRNA sequencing for accurate species identification [10]. Accurate diagnosis is vital for appropriate treatment with antibiotics and guidance on source control in chronic wound patients.

## Case presentation

A 72-year-old Caucasian male with a known history of multiple episodes of lower extremity cellulitis, homelessness, paroxysmal atrial fibrillation with medication noncompliance, and current IV drug use presented to the emergency department (ED) with worsening left lower extremity pain. Other symptoms included worsening nausea, vomiting, diarrhea, and confusion. Symptoms began six days prior to his ED visit. He had recently completed a course of cephalexin provided by a local free clinic, which did not improve his symptoms. At that time, he was recommended to go to the ED for evaluation, but he wished to try oral antibiotic therapy first. Due to the progression of symptoms, he elected to come to the ED for evaluation and treatment.

**Figure 1:**
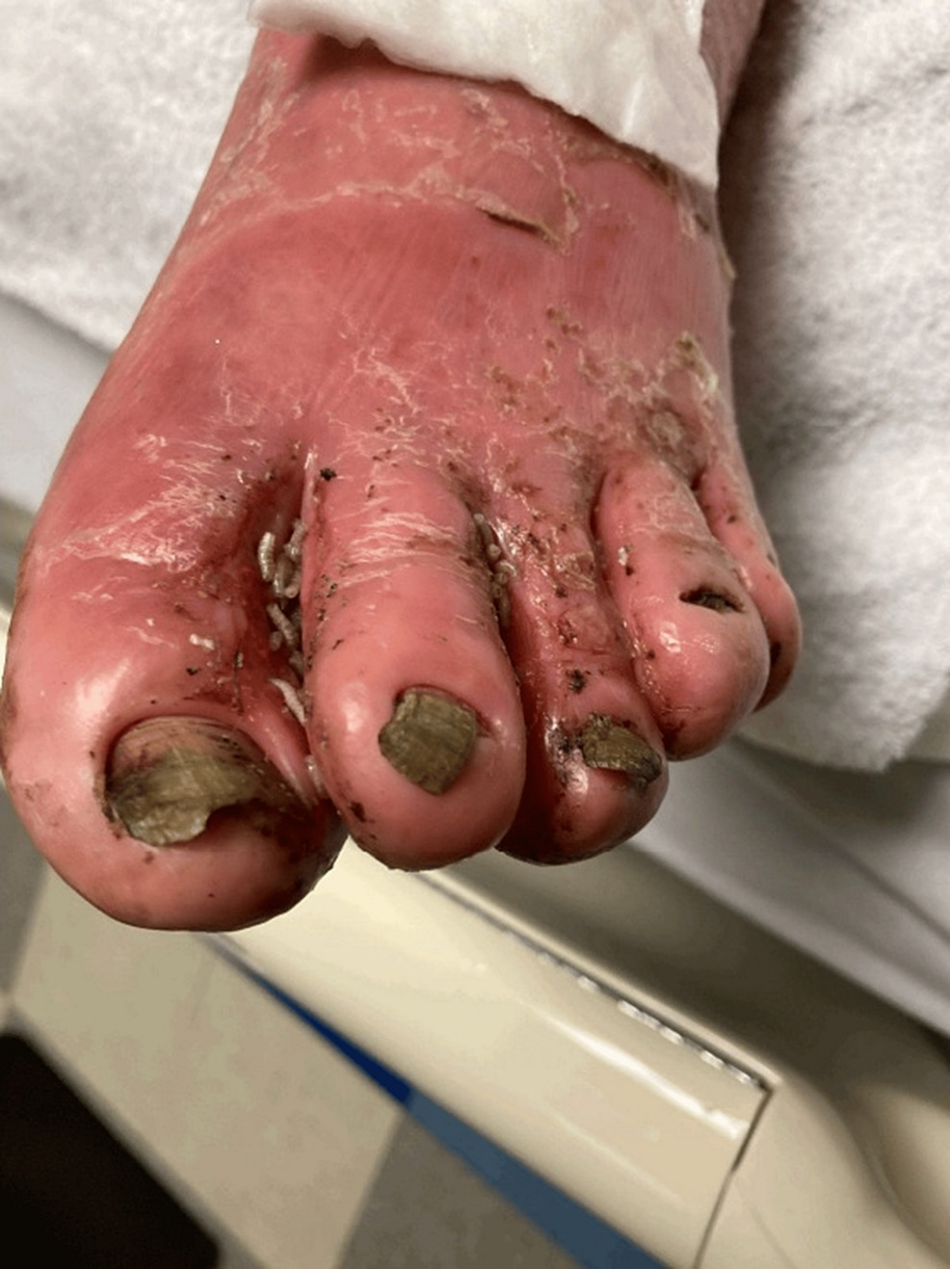
The patient's foot, with visible maggots and chronic wounds

On physical examination, a chronic left foot wound with maggot infestation was observed (Figure 1).

**Figure 2:**
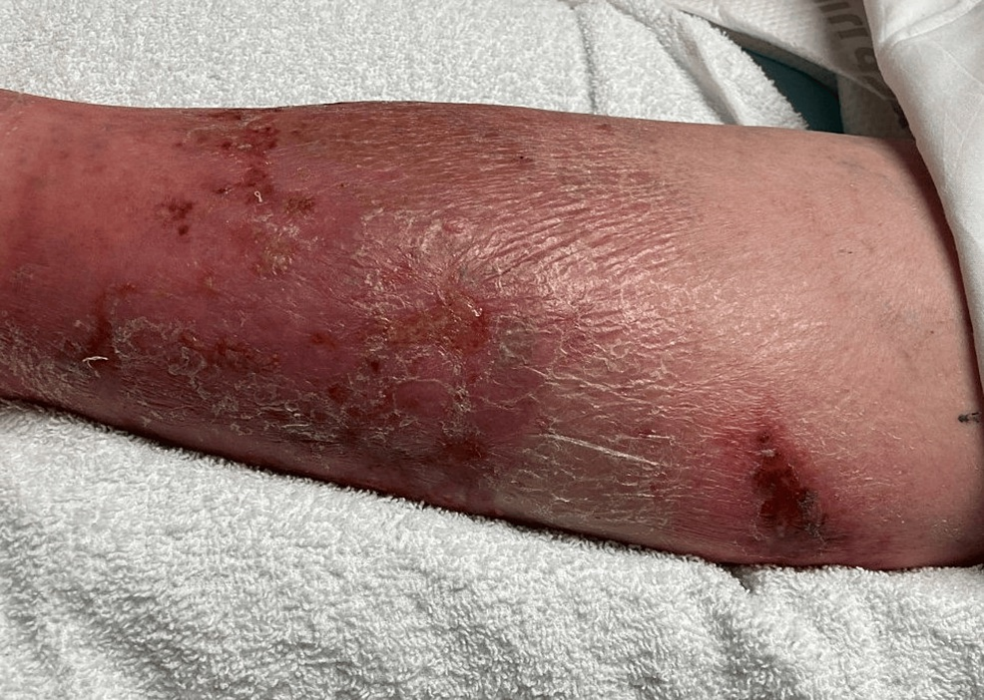
The patient's leg, with physical exam findings consistent with cellulitis

Erythema and swelling extended from the distal foot to directly distal to the knee. The area of redness and swelling was significantly tender to light palpation. A small amount of purulence was appreciated (Figure 2).

The patient had a fever of 101.0°F. Telemetry and follow-up EKG confirmed atrial fibrillation with rapid ventricular rate; the heart rate was sustained at 140-160 beats per minute. The respiratory rate was 22 breaths per minute, and the patient was saturating appropriately on room air. Initially, he was hypotensive with a blood pressure of 69/47; however, he responded appropriately to fluid resuscitation and was normotensive after two liters of lactated Ringer's solution. His Glasgow Coma Scale (GCS) was 14. The patient was following commands appropriately but showed lethargy with occasionally confused responses. His complete blood count (CBC) was significant for an elevated white blood cell count of 18,000 cells/mm^3^, elevated platelet counts at 390,000 cells/uL. The basic metabolic panel was significant for creatinine of 1.48 mg/dL (unclear baseline), and the lactic acid was elevated at 2.5 mg/dL. The C-reactive protein and erythrocyte sedimentation rate were both elevated at 44 mg/dL and 54 mg/dL, respectively. Blood cultures were drawn upon admission. The patient was admitted to the hospitalist service due to concern for worsening cellulitis. A CT imaging of the left lower extremity did not suggest osteomyelitis. Blood cultures were taken upon ED admission, and the patient was started on IV vancomycin and IV piperacillin-tazobactam for broad-spectrum coverage. He was given two IV pushes of metoprolol tartrate, which led to the resolution of the atrial fibrillation. He was transitioned to oral daily metoprolol after admission. The inpatient infectious disease and wound care services were also consulted for evaluation.

After 24 hours, blood cultures initially suggested *Corynebacterium jeikeium*. However, the microbiology lab was not confident in this reading and sent the specimen for review at a tertiary center. The outside facility completed PCR testing and identified the pathogen as *I. indica*, along with completing susceptibilities. Susceptibility testing showed no significant resistance to common antibiotic treatments. Once susceptibilities were reviewed, the patient’s antibiotic regimen was de-escalated to oral levofloxacin.

After five days of antibiotic treatment, the patient had significant improvement in symptoms. Vital signs returned to normal, and the patient's confusion and lethargy resolved. The erythema and swelling of the lower extremity showed near-complete resolution. After fluid resuscitation and IV metoprolol, the patient's rapid ventricular rate resolved, and he converted to normal sinus rhythm. He continued to maintain a normal sinus rhythm and was afebrile for over 24 hours prior to discharge. He was discharged with plans for outpatient wound debridement, continued wound care follow-up, and an outpatient appointment with infectious disease along with another five days of oral levofloxacin.

## Discussion

*Ignatzschineria* is a genus of gram-negative bacteria that is becoming increasingly relevant in the conversation of bacteremia in certain patient populations [7]. Although still rare, an increasing number of case reports document infection by *I. indica* in the setting of maggot-infested wounds [4-6]. This gram-negative, aerobic, nonmotile bacterium is spread through myiasis. The bacteria reside in the digestive tracts of parasitic flies, commonly *Lucilia sericata* and *Wohlfahrtia magnifica* [7]. The symptoms of this infection are typical of bacteremia and cellulitis, with common symptoms such as erythema, warmth, elevated inflammatory markers, and purulent discharge from the wound site [9]. Many documented cases have led to severe infections and sepsis, especially in patients with multiple comorbidities. There are reported cases of *Ignatzschineria* infections in Europe, Canada, and the United States [7-9]. In all these reported cases, risk factors such as poor hygiene, unsanitary living conditions, maggot-infested wounds, or substance abuse are present [7,8]. These infections can lead to significant morbidity, including prolonged hospital stays, increased healthcare costs, and high mortality rates [1,2]. Due to its uncommon prevalence, the bacterium is often misidentified on routine blood cultures [10].

Our case stresses the diagnostic challenges associated with *Ignatzschineria* infections and the risk of misdiagnosis with routine blood cultures. Advanced molecular techniques, such as 16S rRNA sequencing, are often required for accurate diagnosis [10]. In this case, the bacteria were misidentified as *C. jeikeium* and required confirmatory testing to reveal the true pathogen. Given the increased incidence and multiple case reports discussing *Ignatzschineria* bacteremia, we feel this case warrants discussion, in hopes of encouraging prompt consideration for this pathogen in certain patient populations with unclear microbiology findings. Because of situations like this, PCR testing is recommended if clinical suspicion for *Ignatzschineria* is high, or if culture findings are ambiguous [10]. While treatment often includes antibiotic therapy with aminoglycosides, trimethoprim-sulfamethoxazole, or fluoroquinolones, there have been documented cases of resistance to cephalosporins, piperacillin/tazobactam, and carbapenems [6]. Accurate diagnosis and evaluation of susceptibilities are vital for appropriate treatment. Thankfully, the sensitivities of our patient’s blood culture were sensitive to fluoroquinolones, and he was able to be appropriately treated with oral levofloxacin. Our case also stresses the necessity of appropriate wound management and debridement for the prevention of this infection.

## Conclusions

*I. indica*, although rare, poses significant diagnostic challenges in patients with chronic maggot-infested wounds. This case demonstrates the importance of considering pathogens like *Ignatzschineria* in patients with certain risk factors, especially when microbiology results are inconclusive. The misdiagnosis of this pathogen as *C. jeikeium* shows the need for advanced diagnostic tests for the accurate identification of certain pathogens. Effective management involves appropriate antibiotic selection, along with possible debridement and regular monitoring in the clinic. The growing number of case reports discussing *Ignatzschineria* bacteremia indicates a need for improved clinical awareness, and further research is essential to better understand the pathogenesis and optimal treatment approaches.
